# Supplementary material for: Daily Fluctuation of Facial Pore Area, Roughness and Redness among Young Japanese Women; Beneficial Effects of Galactomyces Ferment Filtrate Containing Antioxidative Skin Care Formula
Source: J Clin Med. 2021 Jun 5;10(11):2502. doi: 10.3390/jcm10112502 (PMC8200969; doi:10.3390/jcm10112502)
Supplement: Supplementary file 1 [file jcm-10-02502-s001.zip › jcm-1239928-supplementary.pdf]

Supplementary Figure S1

Baseline period at week -4

GFF treatment at week 4

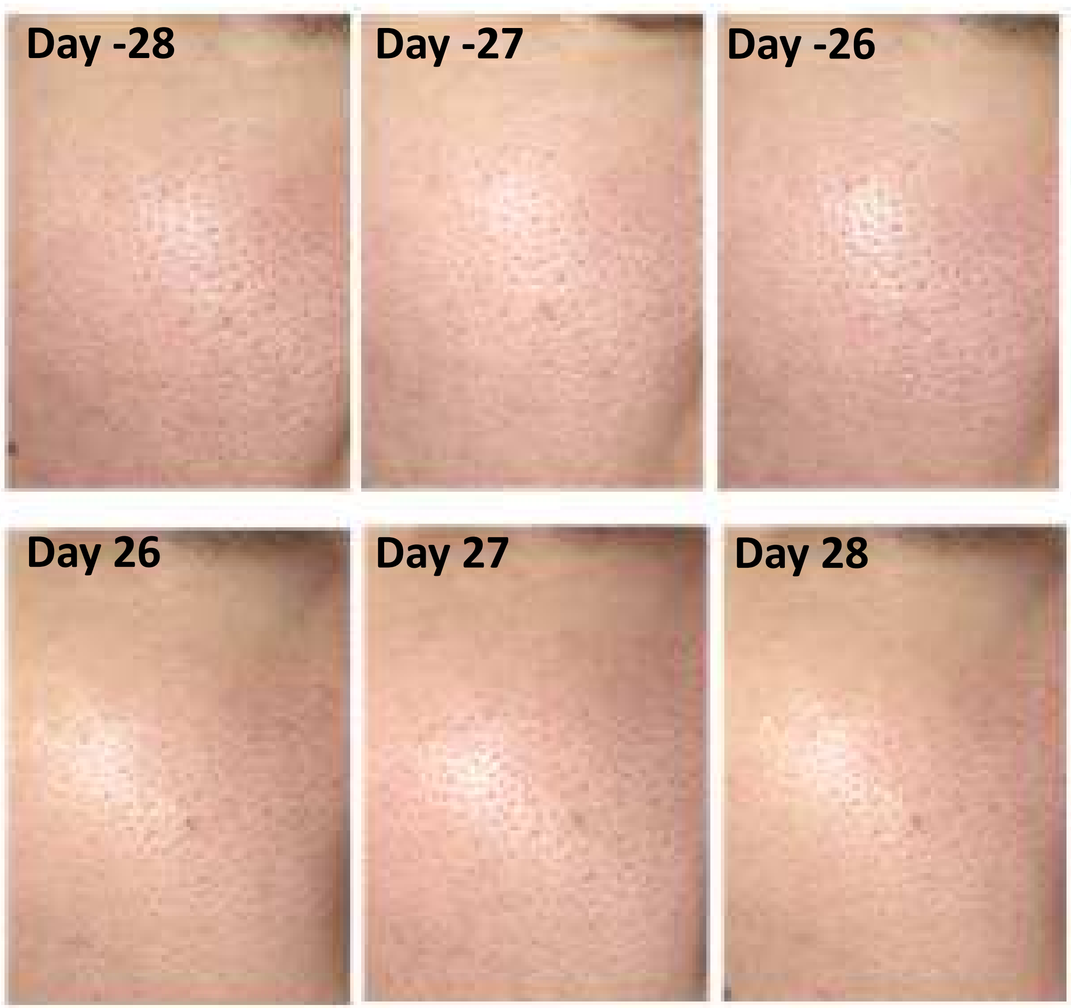


Clinical photos for visible pores.
Visible pores in baseline period at week -4 and in GFF treatment at week 4 are shown.

Supplementary Figure S2

Baseline period
at week -4

GFF treatment
at week 4

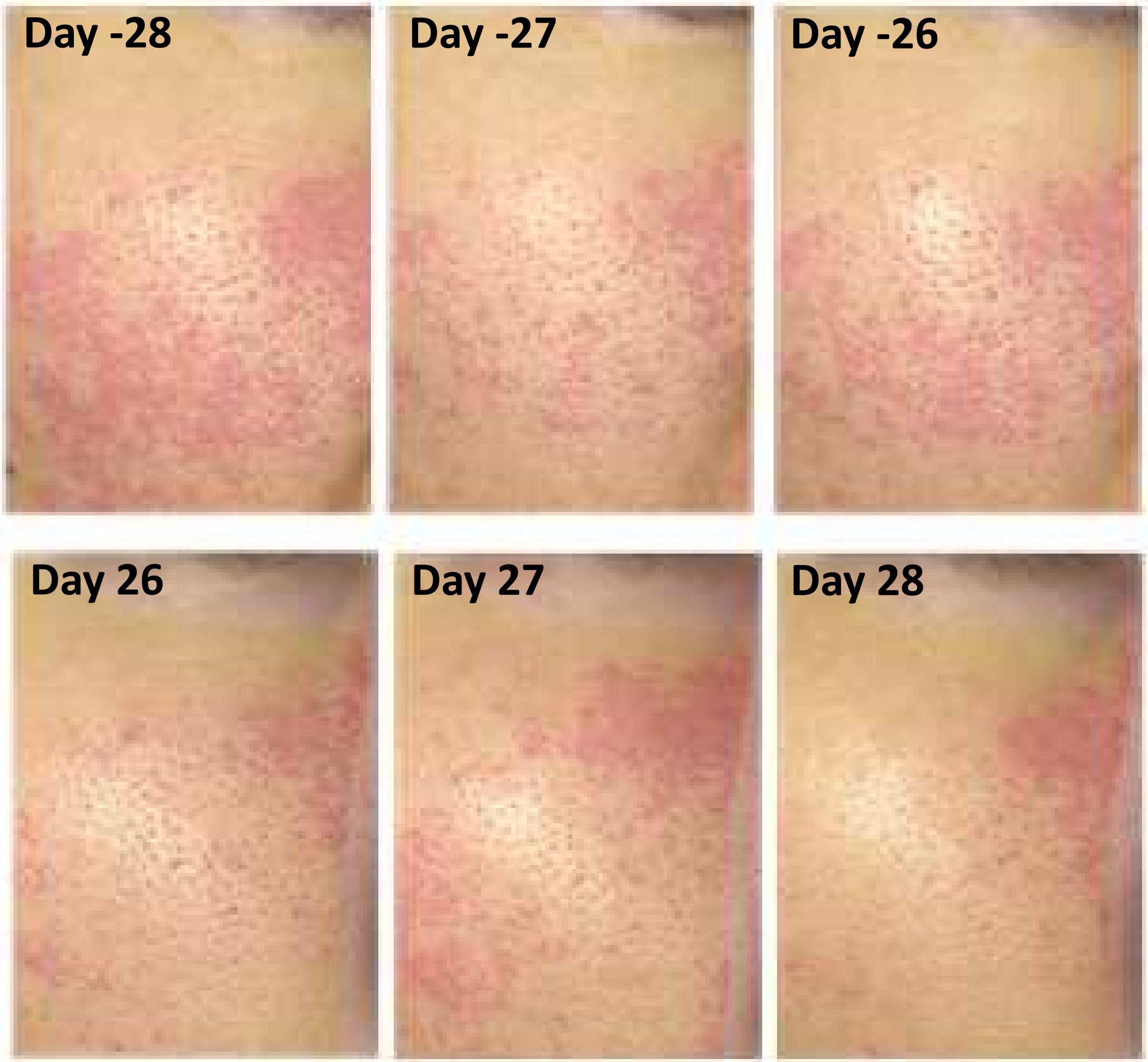


Clinical photos for redness.
Clinical pictures of redness in baseline period at week -4 and in GFF treatment at week 4 are shown.

Supplementary Figure S3

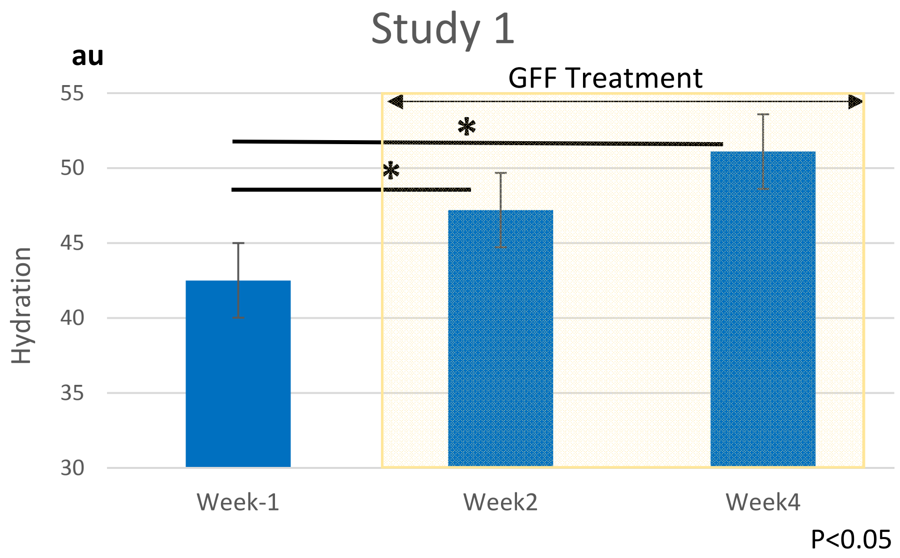


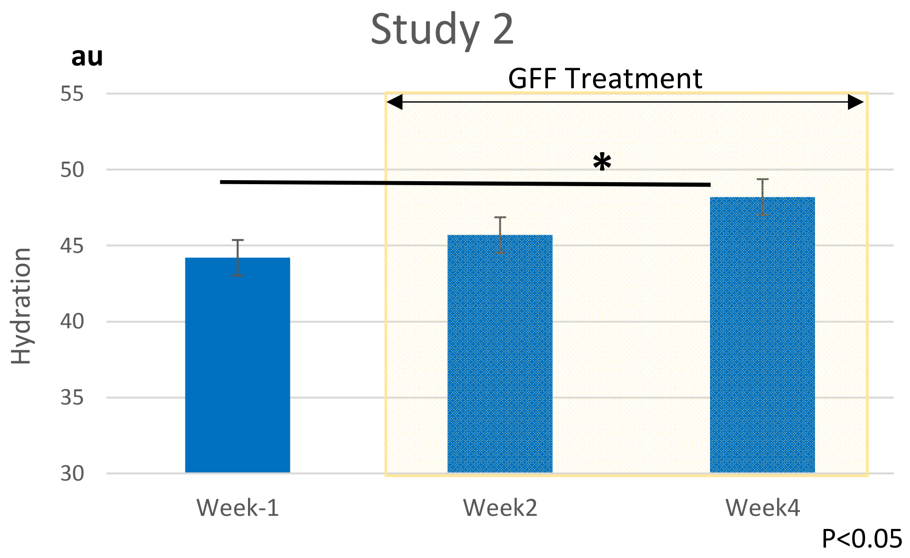


GFF improves facial skin hydration.
Compared with baseline period at week -1, GFF treatment significantly upregulates facial skin hydration at week 2 and week 4 in study 1, and at week 4 in study 2, respectively.

Supplementary Figure S4

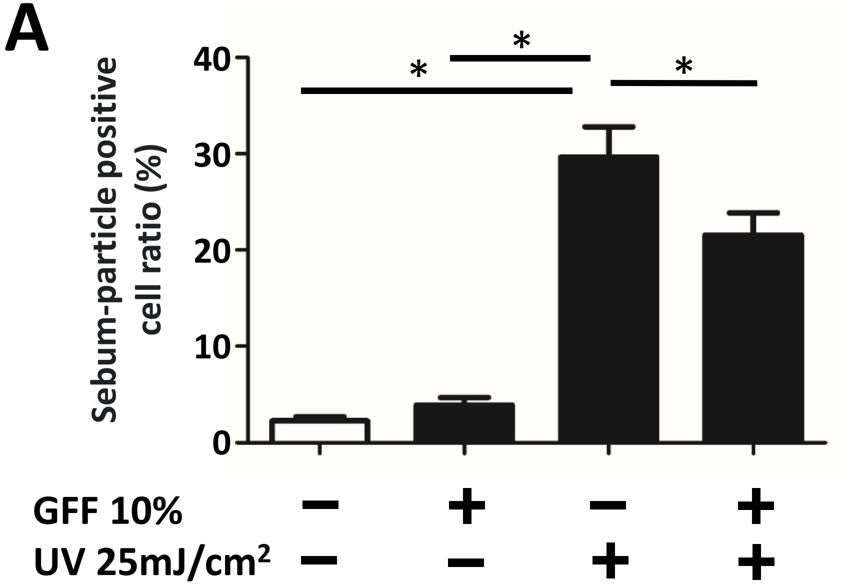


GFF inhibits UVB-induced sebum production by sebocytes.

SEB-1 cells (cultured sebocytes) were treated with or without ultraviolet ray B (UVB) irradiation (25mJ/cm$^2$) in the presence or absence of *Galactomyces* ferment filtrate (GFF, 10%) obtained from P&G Godo Kabusikigaisha. After 24 hrs incubation, sebum production of sebocytes was visualized using an Adipocyte Fluorescence Staining Kit (Cosmo Bio Co., Ltd., Tokyo, Japan).

A: UVB irradiation significantly increased the sebum-particle positive cell ratio compared with non-irradiated control, which was significantly decreased in the combinatory treatment with UVB and GFF.
B: Representative photographs for sebum production. Sebum particles were visualized by fluorescence technique (white arrows).

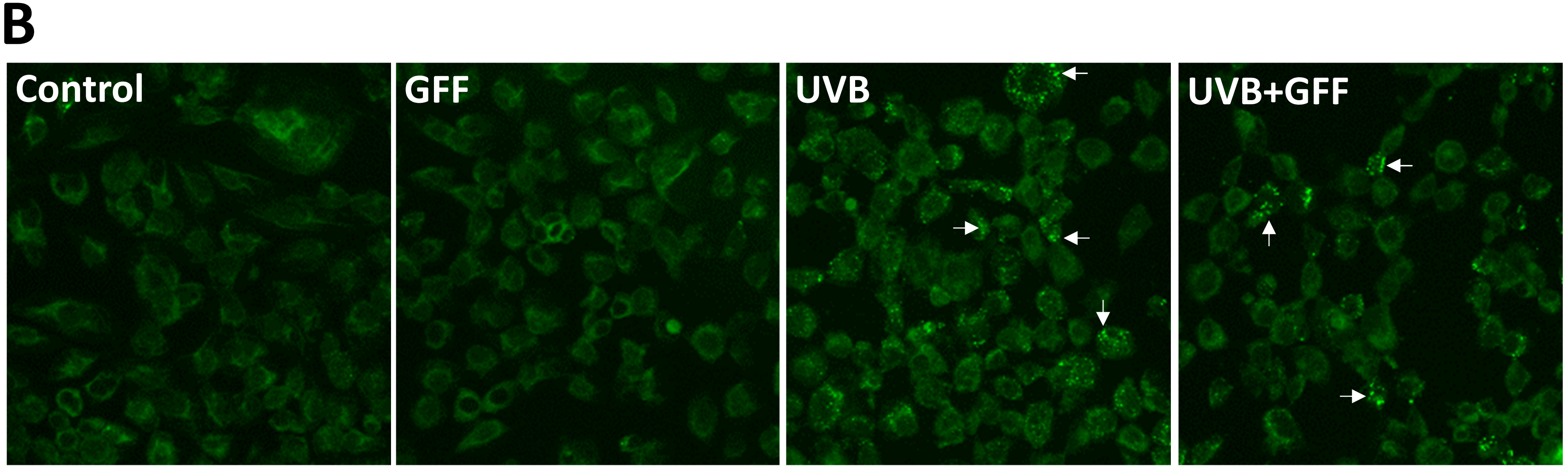

Supplementary Table S1

## Net values of pore area, roughness and redness

| eMR Study 1 | Pore Area | Roughness | Redness |
|---|---|---|---|
| Morning after wake-up | 40.10 (3.829) | 70.20 (2.202) | 59.94 (4.626) |
| Morning after face wash | 34.00 (3.771) | 67.86 (2.213) | 60.96 (4.738) |
| Evening after face wash | 33.81 (3.605) | 68.57 (2.193) | 66.86 (4.927) |
| | | | |
| eMR Study 2 | Pore Area | Roughness | Redness |
| Morning after wake-up | 39.19 (3.978) | 69.55 (2.741) | 58.52 (4.513) |
| Morning after face wash | 33.72 (3.396) | 66.18 (2.478) | 60.28 (4.045) |
| Evening after face wash | 32.67 (3.510) | 67.01 (2.473) | 65.72 (4.051) |

Facial pore area, roughness and redness are evaluated by eMR Pro calculation software. In baseline period at week -4 to week -1, pore area and roughness were largest in the morning after wake-up. Redness was largest in the evening after face wash.  Each value (a.u.) is expressed with mean and (standard deviation).
